# Supplementary material for: Exploring the trade-offs between electric heating policy and carbon mitigation in China
Source: Nat Commun. 2020 Nov 27;11:6054. doi: 10.1038/s41467-020-19854-y (PMC7695859; doi:10.1038/s41467-020-19854-y)
Supplement: Supplementary file 1 — Supplementary Information [file 41467_2020_19854_MOESM1_ESM.pdf]

Supplementary Information

Exploring the Trade-offs between Electric Heating Policy and Carbon Mitigation in China

Jianxiao Wang, Haiwang Zhong, Zhifang Yang, Mu Wang, Daniel M Kammen, Zhu Liu, Ziming Ma, Qing Xia, Chongqing Kang

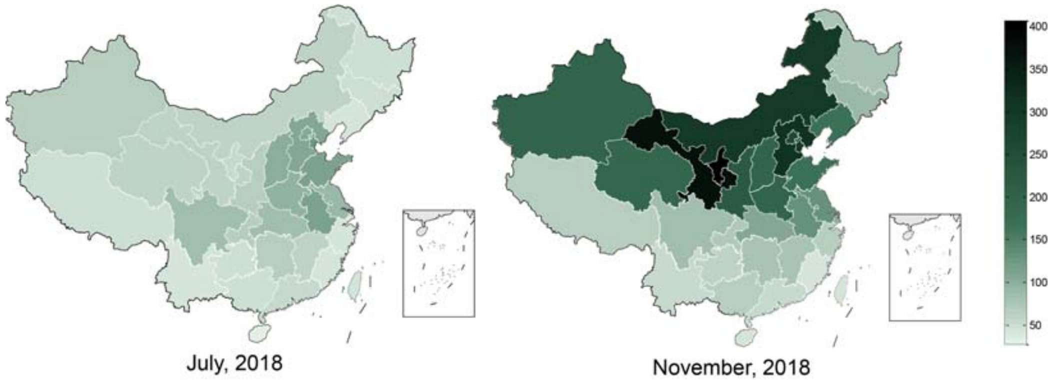

Supplementary Figure 1. China's average air quality index during one day in July and November in 2018.

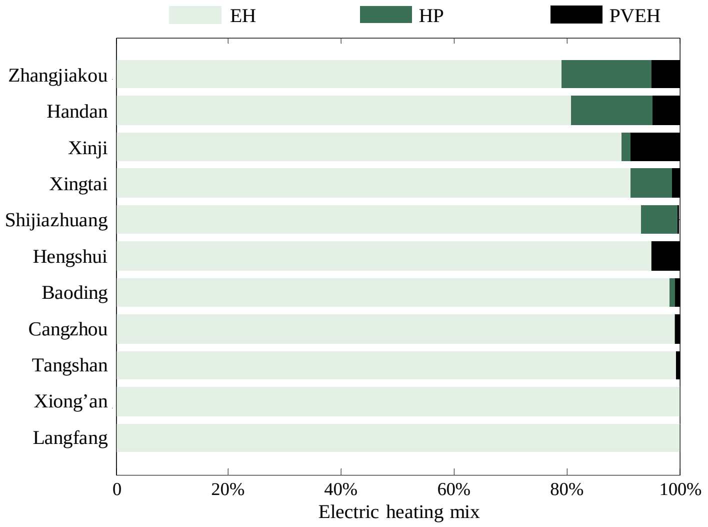

Supplementary Figure 2. Electric heating mix in 11 cities in Hebei province in 2018. EH, HP and PVEH are short for electric heater, heat pump and photovoltaic-powered electric heating.

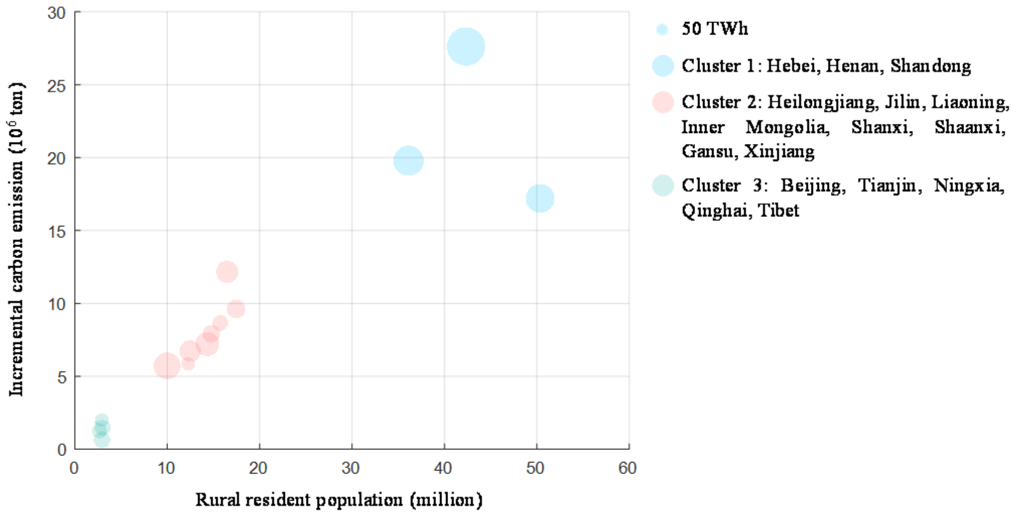

Supplementary Figure 3. Relationship between provincial carbon emission caused by electric heating and rural resident population in Northern China in 2015. Each bubble represents the data of a province, whose radius indicates provincial electricity consumption in 2015.

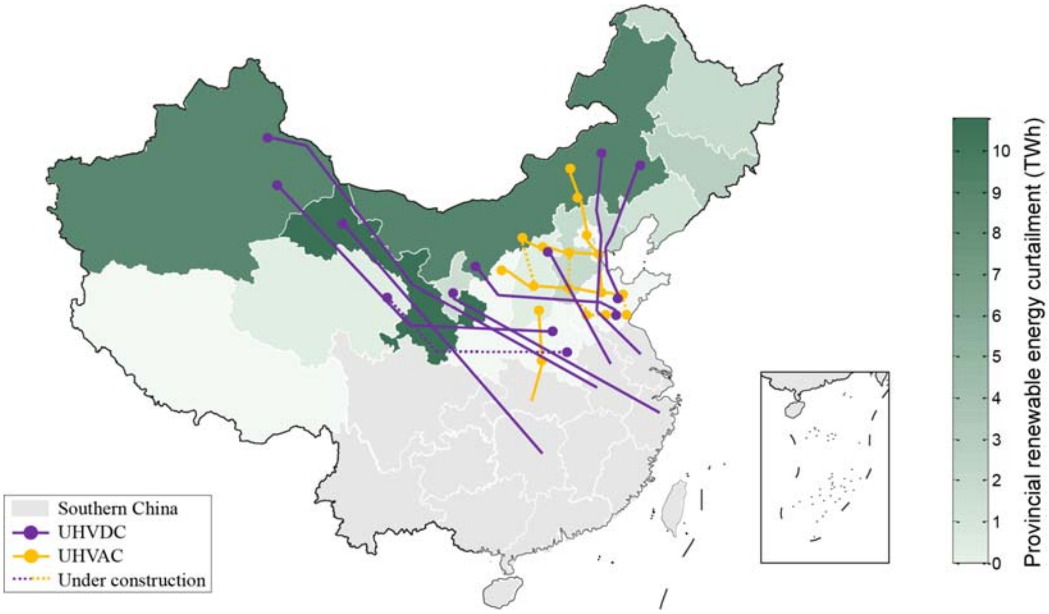

Supplementary Figure 4. Provincial renewable energy curtailment and UHVDC/AC configuration in Northern China in 2015. The dots represent the substations in power sending and receiving provinces. The solid lines represent the operating transmission systems, and the dashed ones represent the transmission networks under construction.

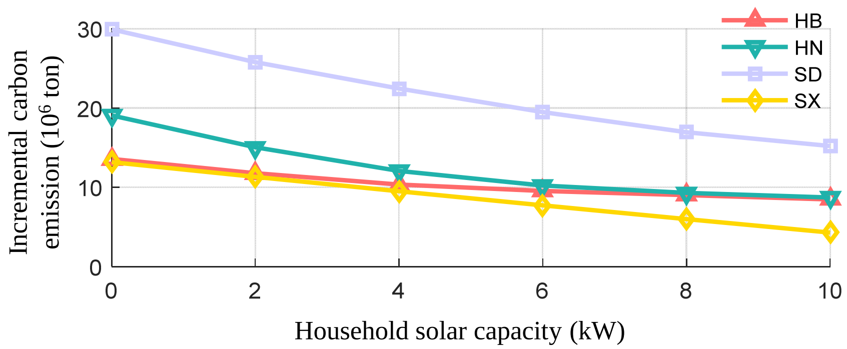

Supplementary Figure 5. Relationship between carbon emission and household installed photovoltaic capacity in Hebei, Henan, Shandong and Shanxi in 2015.

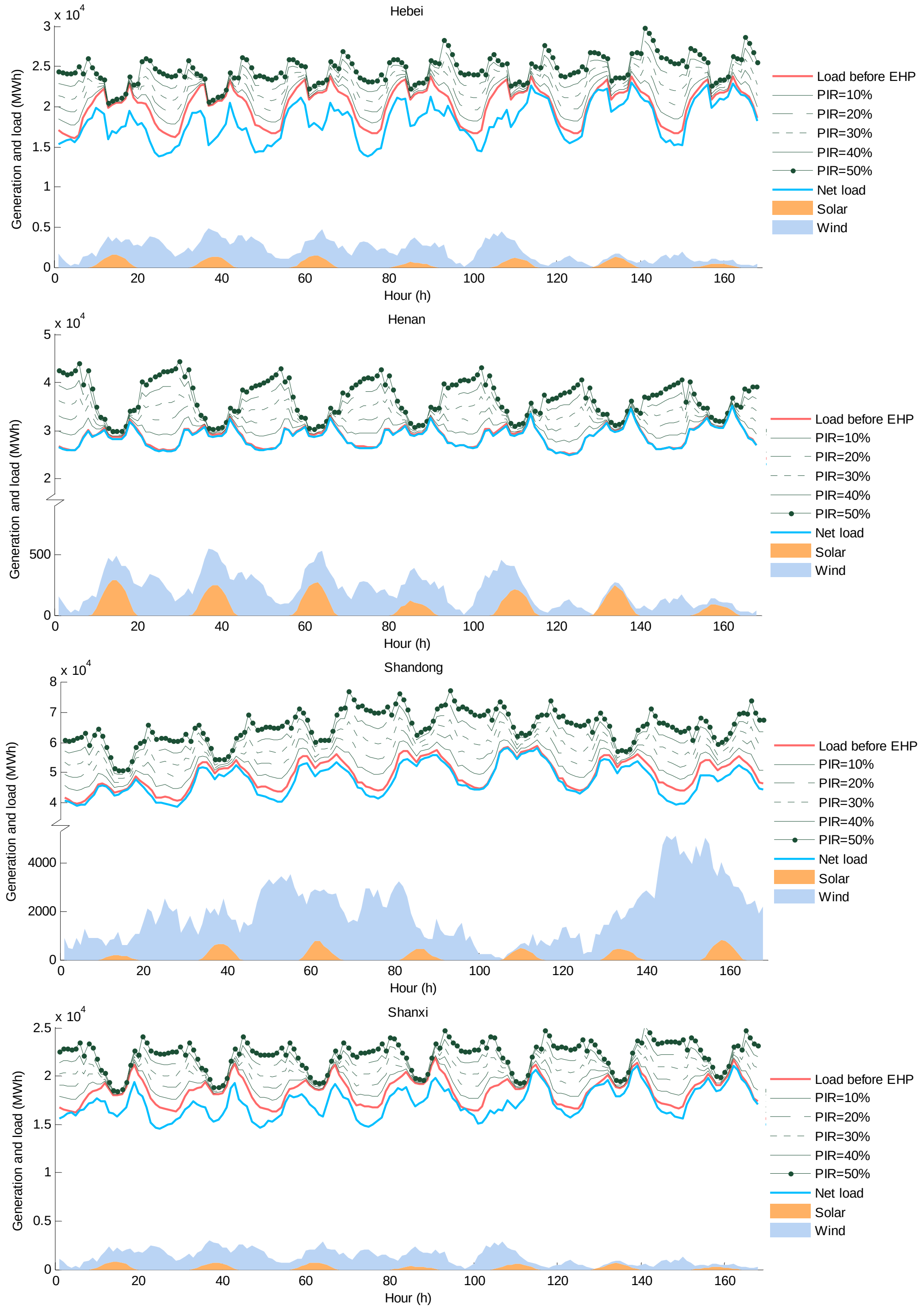

Supplementary Figure 6. Hourly renewable generation and load profiles during one week in Hebei, Henan, Shandong and Shanxi in 2015. “EHP” and “PIR” are short for electric heating policy and policy implementation rate, respectively. Net load equals the difference between load and renewable generation.

[15,30] m^2

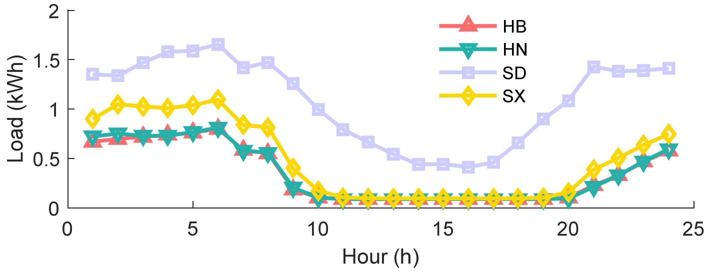

(30,50] m^2

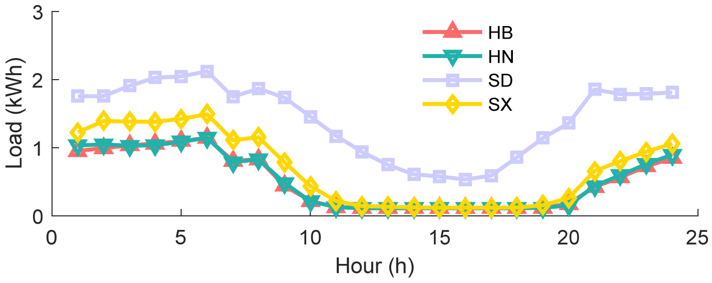

(50,70] m^2

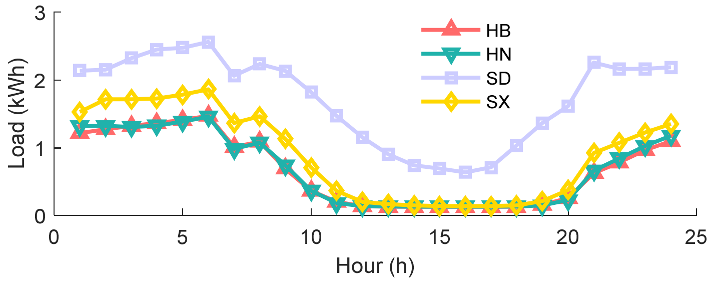

(70,90] m^2

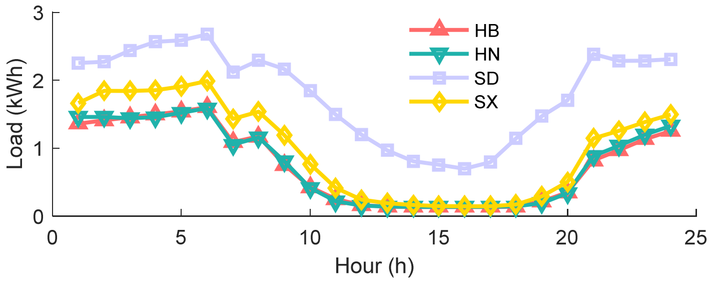

(90,120] m^2

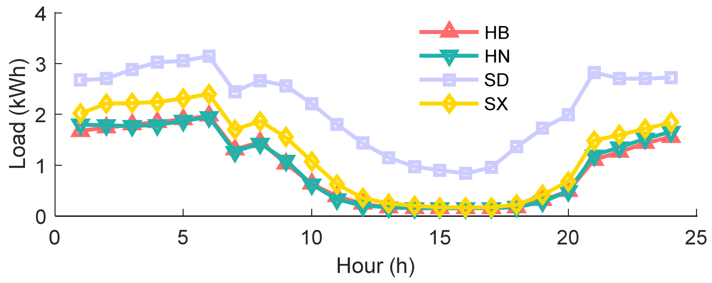

(120,150] m^2

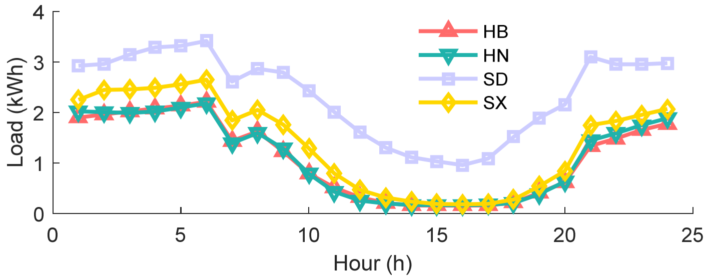

(150,180] m^2

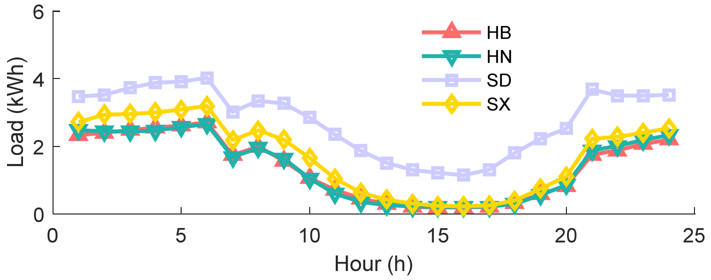

(180,250] m^2

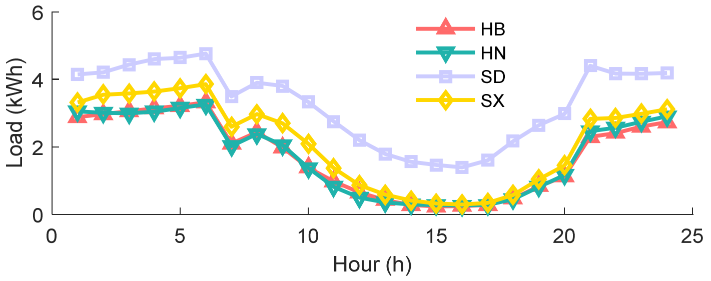

Supplementary Figure 7. Average hourly electric heating load per household during one day in Hebei (HB), Henan (HN), Shandong (SD) and Shanxi (SX) simulated by EnergyPlus.

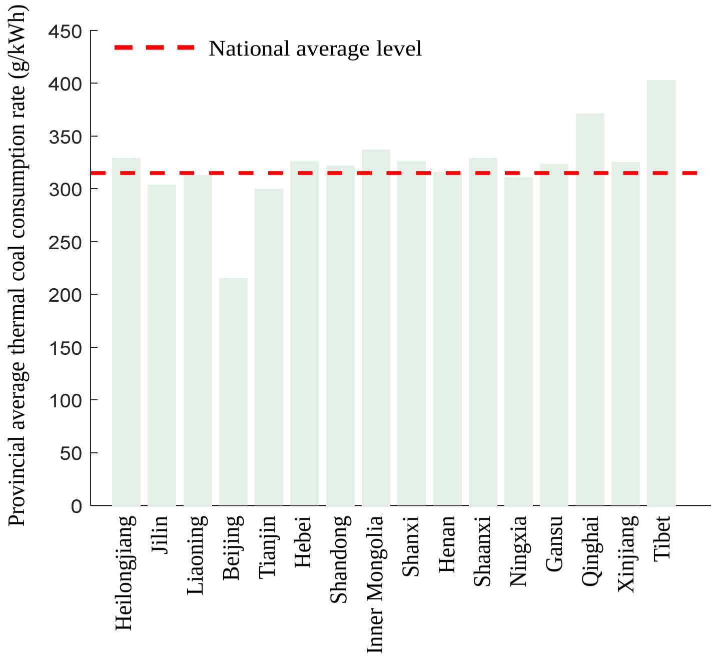

Supplementary Figure 8. Provincial average thermal coal consumption rates in 2015.

Supplementary Tables

Supplementary Table 1 Annual development of various electric heating devices in Hebei in 2018 (Unit: Household)

City	County	Electric heater	Photovoltaic	Others
Shijiazhuang	Wuji	14258	0	0
	Jinzhou	8560	0	0
	Zhaoxian	9021	0	0
	Xinle	4624	0	0
	Xingtang	3663	0	0
	Gaoyi	8293	0	0
	Shenze	4611	0	0
	Yuanshi	6213	0	0
	Lingshou	4906	0	0
	Pingshan	5323	0	0
	Zanhuang	4161	0	0
	Luancheng	0	315	0
	Others	0	0	5000
	Total	73633	315	5000
Zhangjiakou	Chongli	829	208	0
	Zhangbei	876	260	0
	Huailai	1000	0	0
	Xuanhua	800	0	0
	Zhuolu	800	0	0
	Guyuan	500	0	0
	Huaian	406	0	0
	Kangbao	400	0	0
	Shangyi	398	0	0
	Yuxian	322	0	0
	Qiaoxi	302	0	0
	Jingkai	300	0	0
	Qiaodong	236	0	0
	Chicheng	173	0	0
	Yangyuan	150	0	0
	Others	0	0	1495
	Total	7492	468	1495
Chengde	Shuangqiao	0	170	0
	Pingquan	300	0	0
	Chengde	180	0	0
	Shuangluan	200	160	0
	Fengning	200	0	0
	Longhua	100	0	0
	High-tech Zone	100	0	0
	Kuancheng	100	0	0
	Yingzi	10	0	0
	Others	0	0	0
	Total	1190	330	0
Qinhuangdao	Qinglong	0	216	0
	Haigang	0	165	0
	Changli	0	158	0
	Shanhaiguan	0	0	50
	Lulong	0	0	26

	Others	0	0	0
	Total	0	539	76
Tangshan	Yutian	3622	0	0
	Fengnan	2826	0	0
	Qian’an	3178	0	0
	Luannan	3728	245	0
	Laoting	2695	0	0
	Luanxian	2355	0	0
	Fengrun	3533	0	0
	Zunhua	6862	0	0
	Qianxi	2831	0	0
	Caofeidian	2837	0	0
	Guye	2000	0	0
	Others	0	0	0
	Total	36467	245	0
Baoding	Qingyuan	6197	0	0
	Wangdu	2150	0	0
	Anguo	6489	0	0
	Gaobeidian	1049	0	0
	Lixian	1754	0	0
	Quyang	2597	0	0
	Shunping	4846	0	0
	Boye	3511	0	300
	Fuping	0	208	0
	Others	0	0	0
	Total	28593	208	300
Cangzhou	Suning	2004	0	0
	Qingxian	3000	0	0
	Xianxian	140	100	0
	Nanpi	100	0	0
	Xinhua	5682	0	0
	Wuqiao	793	0	0
	Huanghua	470	0	0
	Haixing	1000	0	0
	Botou	620	0	0
	Dongguang	150	0	0
	Yunhe	261	0	0
	Others	0	0	0
	Total	14220	100	0
Hengshui	Zaoqiang	0	630	0
	Wuyi	2992	0	0
	Shenzhou	1673	0	0
	Gucheng	1413	0	0
	Wuqiang	840	0	0
	Raoyang	1187	0	0
	Jingxian	1732	0	0
	Fucheng	1975	0	0
	Binhu	27	0	0
	Others	0	0	0
	Total	11839	630	0
Xingtai	Nanhe	7120	0	0
	Ningjin	5750	0	0
	Renxian	6209	120	100

	Weixian	6218	150	5000
	Longyao	2804	0	0
	Shahe	7917	0	0
	Baixiang	5248	120	300
	Nangong	5576	0	100
	Xingtai	4969	0	0
	Neiqiu	5465	0	100
	Qinghe	4081	166	0
	Development Zone	2568	0	0
	Guangzong	5234	0	0
	Pingxiang	3858	0	0
	Lincheng	1204	243	0
	Linxi	3396	0	0
	Qiaoxi	864	0	0
	Julu	1805	0	0
	Xinhe	578	240	960
	Others	0	0	0
	Total	80864	1039	6560
Handan	Cixian	7132	196	15
	Linzhang	2798	100	1015
	Jinan New Area	1774	0	0
	Cheng’an	3248	110	15
	Fengfeng	4063	145	5015
	Feixiang	0	420	1315
	Wuan	2000	100	2015
	Weixian	4318	300	15
	Daming	2457	400	15
	Guantao	3208	550	15
	Quzhou	5000	100	15
	Hanshan	5672	0	0
	Congtai	4745	0	0
	Jize	3000	100	15
	Qiuxian	0	400	15
	Fuxing	2965	0	0
	Shexian	0	100	15
	Guangping	1000	130	15
	Others	0	0	0
	Total	53380	3151	9510
Xiong’an		5000	0	0
Langfang		82	0	0
Dingzhou		0	850	0
Xinji		6000	583	100
Total		318760	8458	23041
