# Supplementary material for: Exploring the trade-offs between electric heating policy and carbon mitigation in China
Source: Nat Commun. 2020 Nov 27;11:6054. doi: 10.1038/s41467-020-19854-y (PMC7695859; doi:10.1038/s41467-020-19854-y)
Supplement: Supplementary file 3 — Reporting Summary [file 41467_2020_19854_MOESM3_ESM.pdf]

nature research

Corresponding author(s): Haiwang Zhong

Last updated by author(s): Oct 4, 2020

Reporting Summary

Nature Research wishes to improve the reproducibility of the work that we publish. This form provides structure for consistency and transparency in reporting. For further information on Nature Research policies, see our Editorial Policies and the Editorial Policy Checklist.

Statistics

For all statistical analyses, confirm that the following items are present in the figure legend, table legend, main text, or Methods section.

n/a	Confirmed	
☒	☐	The exact sample size (*n*) for each experimental group/condition, given as a discrete number and unit of measurement
☒	☐	A statement on whether measurements were taken from distinct samples or whether the same sample was measured repeatedly
☒	☐	The statistical test(s) used AND whether they are one- or two-sided<br>*Only common tests should be described solely by name; describe more complex techniques in the Methods section.*
☒	☐	A description of all covariates tested
☒	☐	A description of any assumptions or corrections, such as tests of normality and adjustment for multiple comparisons
☐	☒	A full description of the statistical parameters including central tendency (e.g. means) or other basic estimates (e.g. regression coefficient) AND variation (e.g. standard deviation) or associated estimates of uncertainty (e.g. confidence intervals)
☒	☐	For null hypothesis testing, the test statistic (e.g. F, t, r) with confidence intervals, effect sizes, degrees of freedom and P value noted<br>*Give P values as exact values whenever suitable.*
☒	☐	For Bayesian analysis, information on the choice of priors and Markov chain Monte Carlo settings
☒	☐	For hierarchical and complex designs, identification of the appropriate level for tests and full reporting of outcomes
☒	☐	Estimates of effect sizes (e.g. Cohen's *d*, Pearson's *r*), indicating how they were calculated

Our web collection on statistics for biologists contains articles on many of the points above.

Software and code

Policy information about availability of computer code

Data collection	EnergyPlus 8.9.0 is used to collect weather data.
Data analysis	EnergyPlus 8.9.0 is used to analyze building energy consumption. MATLAB R2013b (8.2.0.701) is used to analyze electric power system dispatch.

For manuscripts utilizing custom algorithms or software that are central to the research but not yet described in published literature, software must be made available to editors and reviewers. We strongly encourage code deposition in a community repository (e.g. GitHub). See the Nature Research guidelines for submitting code & software for further information.

Data

Policy information about availability of data

All manuscripts must include a data availability statement. This statement should provide the following information, where applicable:

- Accession codes, unique identifiers, or web links for publicly available datasets
- A list of figures that have associated raw data
- A description of any restrictions on data availability

Outdoor air temperature data are available from the dataset arranged by the World Meteorological Organization ([https://www.energyplus.net/weather]). Solar irradiance and power generation data are available from the National Renewable Energy Laboratory ([https://pvwatts.nrel.gov]). The source data underlying Figs. 1-5 and Supplementary Figs. 1-8 are provided as a Source Data file.

Field-specific reporting

Please select the one below that is the best fit for your research. If you are not sure, read the appropriate sections before making your selection.

☐ Life sciences ☐ Behavioural & social sciences ☒ Ecological, evolutionary & environmental sciences

For a reference copy of the document with all sections, see nature.com/documents/nr-reporting-summary-flat.pdf

Ecological, evolutionary & environmental sciences study design

All studies must disclose on these points even when the disclosure is negative.

Study description	To quantify CO2 induced by China's Electrical Heating Policy (EHP), we propose a theoretical model considering both power generation and rural residential heating sectors. We explore the link between China's EHP and national carbon mitigation, and analyze the key factors leading to the diverse performance of the policy implementation in different regions. To address the incompatibility, we provide policy suggestions for China and other countries with similar situations to facilitate the accommodation of renewable energy and to improve electric heating efficiency.
Research sample	The research samples and data used in this paper are collected from open access websites and publications as well as the projects collaborated with State Grid Corporation of China (SGCC) in Hebei, Henan, Shandong and Shanxi provinces.
Sampling strategy	No sample size calculation method is taken. The data in Hebei, Henan, Shandong and Shanxi provinces (4 out of 16 in Northern China) are collected for the analysis in this paper. We assume that the per capita space heating energy consumption in the four provinces equals that in Northern China. Therefore, our collected dataset is sufficient for the analysis.
Data collection	In this paper, the data regarding the proportions of electric heaters, heat pumps and photovoltaic-powered electric heating are collected by the government in Hebei province based on survey. The hourly electric load data are collected from the database in State Grid Corporation of China via smart meters. Other data are collected from open access websites and publications.
Timing and spatial scale	In Northern China, a heating season generally lasts from November 1st to March 31st in the next year. Due to data limitation, we collect all the hourly data from 11/01/2015 to 12/31/2015 in Hebei, Henan, Shandong and Shanxi provinces. Then a theoretical method is designed to expand the two-month results in the four provinces to the heating-season results in Northern China.
Data exclusions	No data are excluded.
Reproducibility	The code used in this study is available from the authors upon request. The experiment can be successfully repeated.
Randomization	This is not relevant because we do not need to allocate the data into experimental groups.
Blinding	The investigators are blinded during data collection and analysis.

Did the study involve field work? ☐ Yes ☒ No

Reporting for specific materials, systems and methods

We require information from authors about some types of materials, experimental systems and methods used in many studies. Here, indicate whether each material, system or method listed is relevant to your study. If you are not sure if a list item applies to your research, read the appropriate section before selecting a response.

Materials & experimental systems

n/a	Involved in the study
☒	☐ Antibodies
☒	☐ Eukaryotic cell lines
☒	☐ Palaeontology and archaeology
☒	☐ Animals and other organisms
☒	☐ Human research participants
☒	☐ Clinical data
☒	☐ Dual use research of concern

Methods

n/a	Involved in the study
☒	☐ ChIP-seq
☒	☐ Flow cytometry
☒	☐ MRI-based neuroimaging
